# Acute Myeloid Leukemia as the Main Cause of Pancytopenia in Iranian Population 

**Published:** 2017-07-01

**Authors:** Hasan Jalaeikhoo, Seyed Mohammad Hossein Kashfi, Pedram Azimzadeh, Ahmad Narimani, Katayon Gouhari Moghadam, Mohsen Rajaienejad, Mehdi Ariana, Manouchehr Keyhani

**Affiliations:** 1 *AJA Cancer Research Center (ACRC), AJA University of Medical Sciences, Tehran, Iran*; 2 *Gastroenterology and Liver Diseases Research center, Research Institute for Gastroenterology and Liver Diseases, Shahid Beheshti University of Medical Sciences, Tehran, Iran*; 3 *AJA Trauma and Surgery Research Center, AJA University of Medical Sciences, Tehran, Iran*; 4 *Hematology and Oncology Research Center, Vali Asr Hospital, Tehran University of Medical Science, Tehran, Iran*

**Keywords:** Pancytopenia, Acute Leukemia, Myelodysplastic Syndrome, AML

## Abstract

**Background & objective:**

Pancytopenia is the reduction in the number of all 3 major cellular elements of blood and leads to anemia, leukopenia, and thrombocytopenia. A wide variety of etiologies result in pancytopenia including leukemia, aplastic anemia, and megaloblastic anemia. The current study identified the different etiologies of pancytopenia based on bone marrow examination in Iranian patients with pancytopenia.

**Methods:**

A total of 683 cases of pancytopenia with various etiologies were selected for this retrospective study. Bone marrow biopsy was performed with the standard technique using Jamshidi needle. The inclusion criteria for patients with pancytopenia were hemoglobin (Hb) <10 g/dL, total leukocyte count (TLC) <4 x 10^9^/L, and platelet count <140 x 10^9^/L.

**Results:**

In the present study acute leukemia was the first most common etiology detected in 235 (35.4%) patients in which acute myeloid leukemia (AML) comprised the majority of cases 142 (21.4%), followed by myelodysplastic syndrome (MDS) 100 (15%). In patients less than 20 years old, acute leukemia was also the commonest cause identified in 56 (57.7%) cases in which acute lymphoblastic leukemia (ALL) with 38.7% was the most common etiology; however in adults (>45 year old), AML accounted for the majority of cases 76 (53.5%).

**Conclusion:**

Since acute leukemia was the commonest etiology in both young and adults in which AML accounted for the majority of cases with pancytopenia in Iranian population, there was an urgent need to identify the underlying molecular or genetic mechanism of this malignancy for better further medical management and patients` survival.

## Introduction

Pancytopenia is the reduction in the number of all 3 major cellular elements of blood and as a result it leads to anemia, leukopenia and thrombocytopenia (1, 2). A wide variety of etiologies result in pancytopenia including aplastic anemia, megaloblastic anemia, leukemia, etc. The common symptoms and signs consist of fever, fatigue, pallor, weight loss, bleeding, splenomegaly, lymphadenopathy, and hepatomegaly (3). Therefore, when a patient refers with prolonged fever, bleeding and pallor, pancytopenia should be taken under consideration in the etiology of presented disease. The underlying mechanism of pancytopenia development varies in different populations and factors such as age, period of observation, nutritional status, climate, genetic differences, and the prevalence of infections may alter the occurrence rate (4, 5). Pancytopenia can result from aplastic anemia (reduction in hematopoietic stem cell production) or it may arise from infection, infiltration of bone marrow, immune-mediated damage or hypersplenism in which normal cells trapped in hypertrophied and overactive reticuloendothelial system (1, 6). Besides megaloblastic anemia, in the developing countries, infections such as kala azar, enteric fever malaria, and bacterial infections might be the most common reasons of pancytopenia incidence (7). As it is a diagnostic challenge to physician, finding the accurate etiologies of this condition is very important for further clinical management of the cases. The severity of pancytopenia and the underlying mechanism by which the disease appears are the main elements to determine the appropriate management and prognosis of the patients (8). Therefore, bone marrow examination in every suspected individual is crucial for better evaluation of pancytopenia cases (9). The current study aimed at identifying the frequency of different etiologies of pancytopenia based on bone marrow examination and assessing the contribution of gender and age to pancytopenia incidence in Iranian patients with pancytopenia.

## Materials and Methods 

In the current retrospective cohort study, bone marrow examination was performed on 10500 patients referred to AJA Cancer Research Center (ACRC), Imam Reza Hospital for hematological evaluation from 1996 to 2013. A total of 683 cases of pancytopenia with various etiologies were selected for the study. The inclusion criteria for patients with pancytopenia were hemoglobin (Hb) <10 g/dL, total leukocyte count (TLC) <4 x 10^9^/L, and platelet count <140 x 10^9^/L. Cases who had received chemotherapy or immunosuppressive drugs and the ones who had unclear pathology results were excluded from the study. Bone marrow examination was performed on each patient. Full general and systemic examinations were performed on patients and clinical relevant data were recorded. Bone marrow biopsy was performed with the standard technique using Jamshidi needle from posterior iliac crest of the patients under local anesthesia. The staining procedure was performed by standard Wright stain. Cytochemical stains were also applied if required. The current study was approved by the Ethics Committee of the AJA Cancer Research Center (ACRC) at AJA University of Medical Sciences; the study protocol was in accordance with the ethical standards of the Helsinki Declaration.

## Results

A total of 665 cases with pancytopenia were enrolled in the present study (467 male and 198 female). The mean age was 47.60±21.14 years (range, 3 to 95). The final pathological diagnosis in cases of pancytopenia is indicated in Table 1. In the current study, the commonest presentation was fatigue in 30% followed by fever 21.8%, bleeding 15.3%, weight loss 10.8%, bone pain 7.1%, headache dizziness 5.4%, abdominal pain 4.3%, jaundice 2.1%, and short of breath 1.6%. In the present study, acute leukemia was the most common etiology detected in 235 (35.4%) patients followed by myelodysplastic syndrome (MDS). In patients below 20 years old, acute leukemia was the commonest etiology detected in 56 (57.7%) cases, followed by aplastic anemia (AA) in 18 (18.6%) cases. Similar to young patients, in cases over 40 years old, acute leukemia was the most prevalent cause observed in 94 (22.4%) cases followed by MDS 91(21.6%). In young patients below 20 years old with acute leukemia, acute lymphoblastic leukemia (ALL) accounted for the highest frequency 36 (37.1%) cases; however in adults, acute myeloblastic leukemia (AML) was the commonest subtype in 76 (18.1%) cases. The frequency of pancytopenia etiologies, according to the selected age groups, is shown in Table 2. The majority of the patients in the study population were above 40 years old 421 (63.3%). Based on the findings, among 665 individuals with pancytopenia, 15 (2.3) cases had normal pathologic result and did not have any malignancies or related hematological malfunction. Among 235 patients with acute leukemia, 162 (68.9%) were male and 73 (31.1%) female. Based on the current study results, in acute leukemia cases, the commonest etiology among male were AML 89 (19.1%), followed by ALL 73 (15.6%), and megaloblastic anemia 65 (13.9%). AML also had the highest rate of frequency in female cases 53(26.8%). The second most prevalent cause of pancytopenia in females was MDS 37 (18.7%) and megaloblastic anemia 65 (15.7). MDS was also identified in 63 (63%) male and 37 (37%) female cases. Megaloblastic anemia was detected in 65 (67.7%) male and 31 (32.3%) female cases. All of the 13 patients with hairy cell leukemia (HCL) were male and 41 (73.2%) out of 56 non-Hodgkin lymphoma (NHL) cases were male. The frequency of 3 hematological elements in patients with pancytopenia is also presented in Table 3. Mean hemoglobin concentration was 5.90 ± 1.90 g/dl. The leucocyte count was 300–4000/mm^3^ of blood (mean 2633/mm^3^). The platelet count was 3 000–100 000 with the mean of 45.20 ± 38.60 × 10^3^/mm^3^ of blood.

## Discussion

Pancytopenia is defined as reduction in all the 3 formed elements of blood: Red blood cells, white blood cells, and platelets bellow the normal reference range (2). There is a variety in etiologies of pancytopenia in different studies (10-13). In European populations, pancytopenia is mostly observed in hematological malignancies and rarely caused by infection (14). 

**Table 1:** The Etiology of Pancytopenia Based on Biopsy Findings in Patients

Percentage	No. of Patients	Diagnosis	No.
9.2	61	Aplastic anemia	1
14	93	ALL	2
2	13	HCL	3
21.4	142	AML	4
14.4	96	Megaloblastic anemia	5
2.6	17	IDA	6
15	100	MDS	7
8.4	56	Non-Hodgkin lymphoma	8
5.1	34	Multiple myeloma	9
3	20	Myelofibrosis	10
2	13	Metastasis to BM	11
2.3	15	Normal	12
0.2	1	Typhoid	13
0.5	3	Hypersplenism	14
0.2	1	Kala azar	15
%100	665	**Total**	16

ALL, acute lymphoblastic leukemia; HCL, hairy cell leukemia; AML, acute myeloid leukemia; IDA, iron deficiency anemia; MDS, myelodysplastic syndrome

**Table 2:** The Frequency of Pancytopenia Etiologies According to Age Groups

Total	>45	35-45	25-35	<25	Age Group n (%) Etiology
61(100%)	20(32.8%)	8(13.1%)	15(24.6%)	18(29.5%)	Aplastic anemia
93(100%)	18(19.4%)	6(6.5%)	33(35.5%)	36(38.7%)	ALL
13(100%)	12(92.3%)	1(7.7%)	0(0%)	0(0%)	HCL
142(100%)	76(53.5%)	20(14.1%)	26(18.3%)	20(14.1%)	AML
96(100%)	68(70.8%)	3(3.1%)	12(12.5%)	13(13.5%)	Megaloblastic anemia
17(100%)	9(52.9%)	2(11.8%)	2(11.8%)	4(23.5%)	IDA
100(100%)	91(91%)	3(3%)	5(5%)	1(1%)	MDS
56(100%)	51(91.1%)	2(3.6%)	1(1.8%)	2(3.6%)	Non-Hodgkin lymphoma
34(100%)	33(97.1%)	0(0%)	0(0%)	1(2.9%)	Multiple myeloma
20(100%)	17(85%)	1(0.5%)	1(0.5%)	1(0.5%)	Myelofibrosis
13(100%)	11(84.6%)	1(7.7%)	0(0%)	1(7.7%)	Metastasis to BM
15(100%)	12(80%)	0(0%)	3(20%)	0(0%)	Normal
1(100%)	1(100%)	0(0%)	0(0%)	0(0%)	Typhoid
3(100%)	2(66.7%)	0(0%)	1(33.3%)	0(0%)	Hypersplenism
1(100%)	0(0%)	0(0%)	1(100%)	0(0%)	Kalaazar
665(100%)	421(100%)	47(100%)	100(100%)	97(100%)	**Total**

ALL, acute lymphoblastic leukemia; HCL, hairy cell leukemia; AML, acute myeloid leukemia; IDA, iron deficiency anemia; MDS, myelodysplastic syndrome

In a study by Keisu M. et al., on Swedish population, results revealed that patients with pancytopenia should be carefully followed up for AA and MDS (15). A comparison of cause of pancytopenia in different studies is presented in Table 4. To the authors` best knowledge; it is the first study of pancytopenia etiologies with this large sample size. In the current study, the commonest presentation was fatigue in 30%, followed by fever 21.8% and bleeding 15.3%. However, Nafil H. et al. reported that pallor and asthenia were detected in 100% of their study population, but fever was only detected in 30.5% of the cases (16). Similar to the current results, Gayathri et al., (11) and Thakkar et al., observed generalized weakness and fever in the majority of patients(17). In the current study, acute leukemia was the first most common etiology in 235 (35.4%) patients, followed by MDS 100 (15%) and megaloblastic anemia 96 (14.4%). In patients below 20 years old, acute leukemia was the commonest etiology detected in 56 (57.7%) cases, followed by AA in 18 (18.6%) and megaloblastic anemia 13 (13.4%). Similar to young patients, in cases over 40 years old, acute leukemia was the most prevalent cause detected in 94 (22.3%) cases, followed by MDS 91 (21.6%) and megaloblastic anemia 68 (16.2). It is interesting that in young patients below 20 year old with acute leukemia, ALL accounted for the highest frequency 36 (37.1%); however in adults, AML was the commonest subtype in 76 (18.1%) cases. It was demonstrated that ALL frequency was approximately 80% in all childhood leukemia cases and it was also common in adults (18). Another study by Yamamoto et al., on leukemia patients showed that AML frequency was mostly common in adults than children (19). In consistent with the current findings, Jha A. et al., reported that acute leukemia was the commonest hematological malignancy in their study (4).

**Table 3:** The Frequency of Three Haematological Elements in Patients With Pancytopenia

**Total (%)**	No. of Subjects	Range	Parameter
**0.1**	1	<3	**Hemoglobin (%)**
**14.1**	96	3-6	
**43.3**	298	6-9	
**42.5**	290	>9	
**14**	99	<1000	**Total leukocyte count (cells/mm** ^3^ **)**
**58**	396	1000-3000	
**27**	188	>3000	
**17.1**	117	<20 000	**Total platelet count (cells/mm** ^3^ **)**
**28.1**	192	20 000-50 000	
**35.9**	245	50 000-100 000	
**18.9**	129	>100 000	

Another study by Mirzai et al., on Iranian population in 2009 showed that leukemia accounted for the most prevalent disorder detected in 54% of cases (20). From the clinical and molecular point of view, AML is a heterogeneous disease (21). It is demonstrated that the number of new cases of AML is 4.0 per 100 000 individuals per year and the number of related deaths is 2.8 per 100 000 each year (22). The mean age of diagnosis is 67 years (23). Approximately 6500 individuals develop acute leukemia in the United States each year (24) which AML accounts for 30% of the acute leukemia deaths. Several genetic alterations are contributed to AML phenotype, which modifies its survival status (25, 26). The major risk factors are the genetic abnormalities and treatment responses. In this regard, several susceptible genes including *NPM1*, *CEBPA*, and *FLT3* are introduced for clinical practices (27, 28). In contrast to the current study, Hayat et al., revealed that most of the cases in their study had non-malignant disorders and acute leukemia were only detected in 11(12.94%) patients (12). Rangaswamy M et al. also revealed similar findings in leukemia cases (5%)(29). MDS is a heterogeneous set of marrow diseases. It is identified by low peripheral blood counts and a tendency toward progression to AML. Age is considered as a risk factor and males are more at risk of developing MDS than females (30). Macrocytic anemia is very common in patients with MDS and the most prevalent sign is fatigue (31). The accurate diagnosis of pancytopenia is crucial to manage the diseases. In the present study, MDS was the 2nd commonest etiology detected in 100 patients (15%). Among 421 cases over 40 years old, the commonest etiology was MDS 91(21.6%). However, in another study on Iranian patients with pancytopenia, Safaei et al. revealed that the most prevalent cause of pancytopenia was MDS detected in 33% of the cases (10). In line with the current study, Elizabeth P. et al. reported that neoplasia had the highest rate of new onset pancytopenia in adults with 65% of the related cases. Among the neoplastic etiologies in adults, the most prevalent diagnoses were MDS (44%) and AML (31%), respectively(32). They demonstrated that MDS was identified in 25% of adult patients above 50 years, but only in 10% of the adult cases younger than 50 years. In the current study, megaloblastic anemia was the 3rd most common cause of pancytopenia detected in 96 (14.4%) cases. In contrary, Gayathri et al., reported that megaloblastic anemia was the most causes of pancytopenia (74.04%), followed by aplastic anemia (18.26%) in India (11). Similar to the previous study, Bhaskar B Thakkar et al. also revealed that megaloblastic anemia was the most common cause of pancytopenia followed by malaria in 19% with pancytopenia (17). Doshi D et al., in a study on 100 patients with pancytopenia also observed a similar rate in this regard (33). However, Santra G et al., reported that the frequency of megaloblastic anemia was 3.6%, which indicates the low incidence of this etiology in their findings (13). In the current study, aplastic anemia was observed in 61(9.2%) patients. The incidence of this etiology varies in different studies (9, 12, 34). In contrast to the current study, Hayat et al., reported that the commonest cause of pancytopenia was aplastic anemia detected in 35.29% of the cases, followed by megaloblastic anemia in 15 (17.64%), and hypersplenism in 13 (15.29%) cases (12). Santra G et al., reported that AA was the most frequent cause of pancytopenia presented in 20.72% of the cases. They also indicated kala azar as another important cause of pancytopenia with 9% frequency (13). In the present study, hypersplenism, typhoid, and kala azar accounted for 3 (0.5%), 1(0.2%), and 1(0.2%) cases, respectively. Among 665 patients with pancytopenia, these 3 etiologies accounted for 5 (0.9%) of all cases. The current study finding was consistent with those of the previous study by Safaei et al., in Iranian population. They did not reported typhoid or hypersplenism as a cause for pancytopenia, and kala azar was only detected in 3% of the cases (10). The low frequency of latter causes indicates that there might be different etiological patterns based on geographical population. In contrast to the current study, Thakkar et al., reported the frequency of 19% for malaria (2nd most common cause) in Indian population, which seems to be endemic in subcontinent countries. Hypersplenism also was the 3rd most common cause in their study observed in 14% of patients (17). Similar findings in the study by Doshi D et al., on 100 patients with pancytopenia showed infections (20%) and hypersplenism (15%) were the 2nd and 3rd most common causes of pancytopenia (33). The majority of patients in the study were above 40 years old, 421(63.3%) cases. However, the study by Rangaswamy M et al., revealed that most of the patients were in age group of 11-30 years (29). It was observed that 97 (14.6%) cases with pancytopenia were below 20 years old and the most common causes of pancytopenia in this age group was acute leukemia detected in 56 (23.8%) cases followed by aplastic anemia in 18 (29.5%). The current study findings were consistent with those of Khan FS et al., on 279 pancytopenic children from 1 month to 16 years old that revealed acute leukemia was the commonest cause of pancytopenia identified in 32.2% of the cases, followed by aplastic anemia 30.8% (35). However Gupta V et al., demonstrated that among young patients under 18 years old, aplastic anemia was the most prevalent cause of pancytopenia (43%), and the 2nd most common cause was acute leukemia identified in 25% of the cases (5). Another study by Jha A et al. on children reported that the commonest finding was hypoplastic bone marrow (38.1%) (4). In contrast to the current study findings, Pine M et al., reported that the most common diagnoses in pancytopenia patients below 18 years old without cancer in the United States was infections (64%) (36). Among 6000 bone marrow biopsies, 13 patients had HCL; which, all of them presented with pancytopenia. The abnormal cell in hairy cell leukemia cases affects reticuloendothelial system and results in pancytopenia or bone marrow failure (37). The current study identified 13 (2%) patients with bone marrow metastasis. Approximately 8% of pancytopenia cases presented metastases risen from different cancers (38)

**Table 4:** Comparative Studies on Pancytopenia

Second Common Cause	Commonest Cause	No. of Cases	Year	Country	Study
MDS (4.5%)	Hypoplastic anemia (52.7%)	319	1987	Israel and Europe	International agranulocytosis and aplastic anemia study group[26]
Hypoplastic anemia (19%)	Neoplastic (32%)	100	1990	Israel and Europe	Keisu M et al.[27]
Aplastic anemia (7.7%)	Megaloblastic anemia (68%)	202	1992	India	Varma et al.[9]
Megaloblastic anemia (22.3%)	Hypoplastic anemia (29.51%	166	1999	India	Kumar et al.[11]
Megaloblastic anemia (23.64%)	Hypoplastic anemia (29.05%)	148	2007	Nepal	Jha et al.[4]
Acute leukemia (25%)	Aplastic anemia (43%)	105	2008	India	GuptaV et al.[5]
Hypersplenism (11.71%)	Aplastic anemia (22.72%)	111	2010	India	Santra G et al.[15]
Hematological disorders (28%)	Infection (64%)	64	2010	USA	Pine et al.[29]
Aplastic anemia (18.26%)	Megaloblastic anemia(74.04)	104	2011	India	Gayathri et al.[13]
Aplastic anemia (18%)	Megaloblastic anemia (66%)	100	2014	India	BarikS et al.[28]
Megaloblastic anemia (23%)	Myelodysplastic syndrome (33%)	100	2014	Iran	Safaei A. et al.[12]
MDS (15%)	Acute leukemia (35.4%)	665	2014	Iran	Present study

MDS, myelodysplastic syndrome.

## Conclusion

In conclusion, acute leukemia was the most common etiology detected in 235 (35.4%) patients in which AML accounted for the majority of cases with pancytopenia in Iranian population. In patients below 20 years old, acute leukemia was also the commonest cause identified in 56 (57.7%) cases. The high frequency of malignant etiologies such as acute leukemia, especially in young patients (below 20 years old), in Iranian patients with pancytopenia requires urgent diagnosis and management and it would be helpful to detect the underlying mechanism for further evaluation and patients' survival.

## References

[1] DMW Pancytopenia, W.s.C (1993). Aplastic anemia and Pure red cell aplasia. Hematology.

[2] Ishtiaq O, Baqai H Z, Anwer F, Hussain N (2004). Patterns of pancytopenia patients in a general medical ward and a proposed diagnostic approach. J Ayub Med Coll Abbottabad.

[3] Alter B P (1996). Fanconi's anemia and malignancies. Am J Hematol.

[4] Jha A, Sayami G, Adhikari R C, Panta A D, Jha R (2008). Bone marrow examination in cases of pancytopenia. JNMA J Nepal Med Assoc.

[5] Gupta V, Tripathi S, Tilak V, Bhatia B D (2008). A study of clinico-haematological profiles of pancytopenia in children. Trop Doct.

[6] Pizzo PA D A A, Behrman RE, Kleigman RM, Jenson HB (2003). The pancytopenias. Nelson textbook of pediatrics.

[7] Bhatnagar S K, Chandra J, Narayan S, Sharma S, Singh V, Dutta A K (2005). Pancytopenia in children: etiological profile. J Trop Pediatr.

[8] Tilak V, Jain R (1999). Pancytopenia--a clinico-hematologic analysis of 77 cases. Indian J Pathol Microbiol.

[9] Varma N, Dash S (1992). A reappraisal of underlying pathology in adult patients presenting with pancytopenia. Trop Geogr Med.

[10] Safaei A, Shokripour M, Omidifar N (2014). Bone marrow and karyotype findings of patients with pancytopenia in southern iran. Iran J Med Sci.

[11] Gayathri B N, Rao K S (2011). Pancytopenia: a clinico hematological study. J Lab Physicians.

[12] Hayat AS K A, Baloch GH, Shaikh N (2014). Pancytopenia;. study for clinical features and etiological pattern of at tertiary care settings in Abbottabad. Professional Med J.

[13] Santra G, Das B K (2010). A cross-sectional study of the clinical profile and aetiological spectrum of pancytopenia in a tertiary care centre. Singapore Med J.

[14] Ito S, Takada N, Ozasa A, Hanada M, Sugiyama M, Suzuki K (2006). Secondary hemophagocytic syndrome in a patient with methicillin-sensitive Staphylococcus Aureus bacteremia due to severe decubitus ulcer. Intern Med.

[15] Keisu M, Ost A (1990). Diagnoses in patients with severe pancytopenia suspected of having aplastic anemia. Eur J Haematol.

[16] Nafil H, Tazi I, Sifsalam M, Bouchtia M, Mahmal L (2012). [Etiological profile of pancytopenia in adults in Marrakesh, Morocco]. East Mediterr Health J.

[17] Thakkar B B, Bhavsar U N, Trivedi N, Agnihotri A (2013). A Study of Pancytopenia in Adult Patients More than 12 Years of Age in North West Region of Saurashtra. National Journal of Medical Research.

[18] Redaelli A, Laskin B L, Stephens J M, Botteman M F, Pashos C L (2005). A systematic literature review of the clinical and epidemiological burden of acute lymphoblastic leukaemia (ALL). Eur J Cancer Care (Engl).

[19] Yamamoto J F, Goodman M T (2008). Patterns of leukemia incidence in the United States by subtype and demographic characteristics, 1997-2002. Cancer Causes Control.

[20] Mirzai A Z, Hosseini N, Sadeghipour A (2009). Indications and diagnostic utility of bone marrow examination in different bone marrow disorders in Iran. Lab Hematol.

[21] Ley T J, Ding L, Walter M J, McLellan M D, Lamprecht T, Larson D E (2010). DNMT3A mutations in acute myeloid leukemia. N Engl J Med.

[22] SEER Cancer Statistics Factsheets Acute Myeloid Leukemia.

[23] Altekruse S, Kosary C, Krapcho M, Neyman N, Aminou R, Waldron W (2010). SEER cancer statistics review, 1975-2007.

[24] Ries L A G, Smith M A, Gurney J, Linet M, Tamra T, Young J (1999). Cancer incidence and survival among children and adolescents: United States SEER Program 1975-1995. Cancer incidence and survival among children and adolescents.

[25] Dash A, Gilliland D G (2001). Molecular genetics of acute myeloid leukaemia. Best Pract Res Clin Haematol.

[26] Gilliland D G, Tallman M S (2002). Focus on acute leukemias. Cancer Cell.

[27] Dohner H, Estey E H, Amadori S, Appelbaum F R, Buchner T, Burnett A K (2010). Diagnosis and management of acute myeloid leukemia in adults: recommendations from an international expert panel, on behalf of the European LeukemiaNet. Blood.

[28] Chiste M, Vrotsos E, Zamora C, Martinez A (2013). Chronic lymphocytic leukemia/small lymphocytic lymphoma involving the aortic valve. Ann Diagn Pathol.

[29] Rangaswamy M, Prabhu, Nandini N M, Manjunath G V (2012). Bone marrow examination in pancytopenia. J Indian Med Assoc.

[30] Ma X, Does M, Raza A, Mayne S T (2007). Myelodysplastic syndromes: incidence and survival in the United States. Cancer.

[31] Sekeres M A, Schoonen W M, Kantarjian H, List A, Fryzek J, Paquette R (2008). Characteristics of US patients with myelodysplastic syndromes: results of six cross-sectional physician surveys. J Natl Cancer Inst.

[32] Weinzierl E P, Arber D A (2013). Bone marrow evaluation in new-onset pancytopenia. Hum Pathol.

[33] Doshi D, Shah A N, Somani S, Jain A, Jivarajani H, Kothari P (2012). Study of clinical and aetiological profile of 100 patients of pancytopenia at a tertiary care centre in India. Hematology.

[34] Incidence of aplastic anemia (1987). the relevance of diagnostic criteria By the International Agranulocytosis and Aplastic Anemia Study. Blood.

[35] Khan F S, Hasan R F (2012). Bone marrow examination of pancytopenic children. J Pak Med Assoc.

[36] Pine M, Walter A W (2010). Pancytopenia in hospitalized children: a five-year review. J Pediatr Hematol Oncol.

[37] Arcaini L, Zibellini S, Boveri E, Riboni R, Rattotti S, Varettoni M (2012). The BRAF V600E mutation in hairy cell leukemia and other mature B-cell neoplasms. Blood.

[38] Brochamer W L, Keeling M M (1977). The bone marrow biopsy, osteoscan, and peripheral blood in non-hematopoietic cancer. Cancer.

